# SDF-1α promotes subchondral bone sclerosis and aggravates osteoarthritis by regulating the proliferation and osteogenic differentiation of bone marrow mesenchymal stem cells

**DOI:** 10.1186/s12891-023-06366-1

**Published:** 2023-04-10

**Authors:** Zhiqiang Meng, Lujun Xin, Bosheng Fan

**Affiliations:** 1 Jiaozuo Coal Industry (Group) Co. Ltd, Central Hospital, No. 1 Jiankang Road, Jiefang District, Jiaozuo, 454000 Henan China; 2 General Hospital of Ningxia Medical University, Ningxia Medical University, Ningxia, China

**Keywords:** Osteoarthritis, Stromal cell-derived factor-1α, Bone marrow mesenchymal stem cells, Subchondral bone, Bone sclerosis

## Abstract

**Background:**

Subchondral bone sclerosis is a major feature of osteoarthritis (OA), and bone marrow mesenchymal stem cells (BMSCs) are presumed to play an important role in subchondral bone sclerosis. Accumulating evidence has shown that stromal cell-derived factor-1α (SDF-1α) plays a key role in bone metabolism-related diseases, but its role in OA pathogenesis remains largely unknown. The purpose of this study was to explore the role of SDF-1α expressed on BMSCs in subchondral bone sclerosis in an OA model.

**Methods:**

In the present study, C57BL/6J mice were divided into the following three groups: the sham control, destabilization of the medial meniscus (DMM), and AMD3100-treated DMM (DMM + AMD3100) groups. The mice were sacrificed after 2 or 8 weeks, and samples were collected for histological and immunohistochemical analyses. OA severity was assessed by performing hematoxylin and eosin (HE) and safranin O-fast green staining. SDF-1α expression in the OA model was measured using an enzyme-linked immunosorbent assay (ELISA), quantitative real-time polymerase chain reaction (q-PCR), and immunohistochemistry. Micro-CT was used to observe changes in subchondral bone in the OA model. CD44, CD90, RUNX2, and OCN expression in subchondral bone were measured using q-PCR and immunohistochemistry. In vitro, BMSCs were transfected with a recombinant lentivirus expressing SDF-1α, an empty vector (EV), or siRNA-SDF-1α. Western blot analysis, q-PCR, and immunofluorescence staining were used to confirm the successful transfection of BMSCs. The effect of SDF-1α on BMSC proliferation was evaluated by performing a CCK-8 assay and cell cycle analysis. The effect of SDF-1α on the osteogenic differentiation of BMSCs was assessed by performing alkaline phosphatase (ALP) and alizarin red S (ARS) staining. Cyclin D1, RUNX2 and OCN expression were measured using Western blot analysis, q-PCR, and immunofluorescence staining.

**Results:**

SDF-1α expression in the DMM-induced OA model increased. In the DMM + AMD3100 group, subchondral bone sclerosis was alleviated, OA was effectively relieved, and CD44, CD90, RUNX2, and OCN expression in subchondral bone was decreased. In vitro, high levels of SDF-1α promoted BMSC proliferation and increased osteogenic differentiation. Cyclin D1, RUNX2, and OCN expression increased.

**Conclusion:**

The results of this study reveal a new molecular mechanism underlying the pathogenesis of OA. The targeted regulation of SDF-1α may be clinically effective in suppressing OA progression.

**Supplementary Information:**

The online version contains supplementary material available at 10.1186/s12891-023-06366-1.

## Background

Osteoarthritis (OA) is a chronic, progressive disease involving articular cartilage, subchondral bone, synovium, and surrounding ligaments [1]. Middle-aged and elderly people often suffer from OA, joint pain, swelling, deformity, and limited mobility year around, and these symptoms are debilitating and seriously affect the quality of life [2]. However, effective treatments or drugs that alleviate the development of OA are currently unavailable [3]. Therefore, the pathogenesis of OA and strategies to prevent this disease have become research hotspots. Studies have shown that structural and functional changes in subchondral bone are important pathological changes involved in OA [4]. When a joint experiences abnormal external mechanical stress, abnormal remodeling of subchondral bone leads to an increased subchondral bone density and increased bone sclerosis, which impairs its ability to absorb stress and cushion shocks, leading to the degeneration of the overlying cartilage and exacerbation of OA progression [5]. Therefore, a deeper understanding of the pathological changes in subchondral bone that occur during OA might help researchers further elucidate the pathogenesis of OA and provide new ideas for the prevention and treatment of OA.

Subchondral bone sclerosis results from the excessive proliferation of osteoblasts and mineralization of the bone matrix [6]. Bone marrow mesenchymal stem cells (BMSCs) have strong self-renewal and multidirectional differentiation potential and play an important role in bone metabolism. Under certain conditions, BMSCs differentiate into osteoblasts and maintain the ability to repair bone in the context of osteonecrosis [7]. BMSCs are derived from bone marrow tissue, and the specific markers CD44, CD71, CD90, and CD105 are often expressed on the surface of these cells [8]. BMSCs, the most commonly used seed cells in bone tissue engineering, have immunomodulatory and anti-inflammatory effects and regulate the local microenvironment by interacting with various secreted cytokines to play a role in repairing damaged tissues [9]. However, few studies have explored the role of BMSCs in the pathogenesis of OA.

Stromal cell-derived factor-1 (SDF-1) is a chemokine with a relatively low molecular weight [10]. Two SDF-1 isoforms have been identified: SDF-1α and SDF-1β. SDF-1α is widely distributed in human tissues and organs and is produced and secreted by osteoblasts, fibroblasts and endothelial cells [11]. SDF-1α is also the only cytokine that binds the receptor CXCR4 to exert its effect. Studies have shown that SDF-1α/CXCR4 signaling participates in the mobilization and migration of stem cells and plays a regulatory role in various diseases [12]. Studies have confirmed that SDF-1α is expressed at high levels in fracture and bone injury sites; thus, BMSCs that express CXCR4 are chemotactically recruited to the injury site and participate in bone repair [13]. However, few studies have investigated the role of SDF-1α in the pathogenesis of OA.

Based on previous studies, we speculate that SDF-1α-treated BMSCs play an important role in the pathogenesis of OA. In this study, a destabilization of the medial meniscus (DMM)-induced OA model was constructed, and SDF-1α expression in the OA model was evaluated. The CXCR4 receptor antagonist AMD3100 was used to block SDF-1α, and changes in subchondral bone and the expression of BMSCs in subchondral bone in the OA model were observed. In vitro, we observed the effect of SDF-1α on the proliferation and osteogenic differentiation of BMSCs. The purpose of this study was to determine the role of SDF-1α in the pathogenesis of OA and clarify the underlying mechanism.

## Materials and methods

### Experimental animals

Eight-week-old healthy adult male wild-type C57BL/6J mice (SPF grade) weighing 20–25 g were obtained from the Laboratory Animal Center of Ningxia Medical University (Yinchuan, China, lot number: SCXK (Jing) 2016-0006). All mice were housed in an SPF environment at a temperature of 22 ± 1 °C on a 12-hour light/dark cycle and provided free access to food and water. The research plan and protocols were approved by the Ethics Committee of Ningxia Medical University (IACUC-NYLAC-2020-069). All mice were divided into the sham group, DMM group, and DMM + AMD3100 group, with 20 mice per group. Ten mice from each group were sacrificed 2 and 8 weeks after the operation for follow-up experiments. All animal experiments were conducted under the standard principles of animal experiment ethics and complied with the Declaration of Helsinki, and the study is reported in accordance with ARRIVE guidelines.

### Establishment of an OA model by DMM

The mice were weighed and intraperitoneally injected with 0.2% sodium pentobarbital (1.0 mL/100 g). After the mice were anesthetized, the skin on the right knee joint was prepared and sterilized, and the mice were placed in the supine position. A 1-cm midline incision was generated, the skin and tissue were removed, the patella was externally dislocated, the joint cavity was exposed, and the tibial ligament of the medial meniscus was cut. The anterior horn of the medial meniscus was exposed, the joint cavity was flushed, the knee joint was reset, and the incision was sutured with 5 − 0 absorbable sutures.

In the sham operation group, the joint capsule was opened, but the tibial ligament of the medial meniscus was not cut.

### Drug intervention

AMD3100 is a CXCR4 antagonist that competitively binds CXCR4 and blocks its interaction with SDF-1α, thereby interfering with the SDF-1α/CXCR4 signaling pathway. The mice in the DMM + AMD3100 group were intraperitoneally injected with AMD3100 (2.5 mg/kg, MCE) daily until sample collection.

### Collection of blood from the heart and serum isolation

After the mice were successfully anesthetized, they were placed in the supine position, and a 1 mL syringe was inserted under the xiphoid process at a 30–45° angle. Once breakthrough was felt, needle insertion was stopped. If blood was aspirated, 800 µL -1000 µL of blood were drawn slowly, incubated at room temperature for 1 h, and centrifuged at 2000–3000 × g for 15 min, and the supernatant was collected and stored at -80 °C until subsequent analysis.

### Tissue extraction and slice preparation

The mice in each group were anesthetized and euthanized by cervical dislocation at the appropriate time point. The skin and muscle were removed from the right lower limb was stripped, the right knee joint was exposed, the joint capsule remained intact, and the entire joint was removed. Ten samples were collected from each group; 5 of these samples were stored at -80 °C until the subsequent analysis, and the other 5 samples were fixed with 4% paraformaldehyde for 2 days and used for CT. After fixation, the samples were allowed to recover and immersed in an EDTA solution for 2 weeks, with the solution changed every 3 days. Then, the samples were dehydrated, cleared, soaked in paraffin overnight, embedded in a paraffin block the next day, and sliced along the sagittal plane of the knee joint at a thickness of 4–5 μm. The sections were observed under a microscope after staining.

### Hematoxylin and eosin (HE) staining

The slices were warmed at 65 °C for 60 min, dewaxed with xylene and ethanol, stained with hematoxylin for 5 min, differentiated with hydrochloric acid-alcohol for 5 s, rinsed until the tissue appeared blue, stained with eosin for 3 min, dehydrated with ethanol and xylene, cleared, dried after mounting with neutral gum, observed and imaged.

### Safranin O-fast green staining

The sections were warmed at 65 °C for 60 min, dewaxed with xylene and ethanol, stained with hematoxylin for 5 min, differentiated in hydrochloric acid-alcohol for 5 s, washed until the tissue appeared blue, stained with a 0.3% fast green solution for 3 min, soaked in 1% acetic acid (glacial acetic acid) for 5 s, stained with 0.2% safranin O dye for 5 min, dehydrated in ethanol, cleared in xylene, sealed with neutral gum, dried, observed and imaged.

### OARSI scoring system

After safranin O-fast green staining, cartilage was stained red, bone tissue was stained green, and cell nuclei were stained blue. Safranin O-fast green staining was used to objectively evaluate the changes in cartilage and subchondral bone. The severity of OA was quantitatively analyzed by scoring the degree of these changes using the OARSI scoring system. Scoring was performed by three independent people, and 5 slices per sample were observed. The final OARSI score was determined by averaging the scores of all slices. A positive correlation was observed between the OARSI score and the severity of OA (Table 1). In this table, 0 is normal, 1–2 represents mild OA, 3–4 represents moderate OA, and 5–6 represents severe OA.

**Table 1:** OARSI scoring criteria

0	Normal cartilage
0.5	Reduction in safranin staining of cartilage without morphological changes
1	Fibrotic changes in the cartilage surface without defects
2	Obvious cracks and loss of cartilage on the cartilage surface with on changes to the calcified cartilage layer
3	Obvious defects in the calcified cartilage layer accounting for less than 25% of this layer
4	Obvious defects in the calcified cartilage layer accounting for 25–50% of this layer
5	Obvious defects in the calcified cartilage layer accounting for 50–75% of this layer
6	Obvious defects in the calcified cartilage layer accounting for more than 75% of this layer

### Enzyme-linked immunosorbent assay (ELISA)

The SDF-1α levels in the mouse serum were determined using an ELISA kit (Abcam, USA) according to the manufacturer’s instructions. The samples to be tested were placed in a plate containing the antibody and incubated for 90 min at 37 °C. Then, the prepared biotinylated antibody was added to the antibody plate, and the sample was incubated at 37 °C in the dark for 1 h. The enzyme conjugate solution was added to the plate, and the sample was incubated in the dark for 30 min at 37 °C. Then, substrate reaction solution was added to the plate, the sample was incubated at 37 °C for 20 min, stop solution was added, and the absorbance was measured at 450 nm. A standard curve was established based on the obtained results, and a statistical analysis was performed.

### Micro-CT analysis

The right knee joints of the mice were fixed with 4% paraformaldehyde for 48 h, and then micro-CT was performed using a SkyScan 1076 instrument (Bruker, Belgium). The scanning conditions were as follows: 80 KV, 300 mA, and a resolution of 12 μm. DataViewer software was used to analyze the parameters of the subchondral bone area and collect the data. The bone volume fraction (BV/TV (%)), trabecular bone separation (Tb. Sp), and bone mineral density (BMD) were measured using Ctvol software to process the results and construct a three-dimensional model of the subchondral bone.

### Immunohistochemical staining

Paraffin sections were warmed at 65 °C for 1 h, dewaxed with xylene and ethanol, subjected to antigen retrieval by the dropwise addition of 0.25% trypsin and incubated at 37 °C for 30 min. Peroxidase blocking solution was added dropwise, and the sections were incubated for 15 min. Then, the goat serum working solution was added dropwise to the tissue for blocking, and the sections were incubated at 37 °C for 30 min. SDF-1α (ab9797, 1:100; Abcam), CD44 (ab189524, 1:50, Abcam), CD90 (DF6849, 1:50, Affinity), RUNX2 (ab23981, 1:50, Abcam), and OCN (DF12303, 1:50, Affinity) primary antibodies were added dropwise to the tissues until they were fully covered, and the sections were incubated at 4 °C for 12 h. The samples were heated at 37 °C for 30 min, incubated with DV-9001 for 30 min at 37 °C, and incubated with a secondary antibody for 60 min at 37 °C. DAB was added for color development, and then the sections were stained with hematoxylin for 5 min, differentiated with hydrochloric acid-alcohol for 5 s and rinsed with tap water until the tissue appeared blue. The sections were dehydrated in ethanol, cleared in xylene, mounted with neutral gum, allowed to dry naturally, observed and imaged.

### Extraction of total RNA and quantitative real-time polymerase chain reaction (qPCR)

Animal tissues or cells were lysed with TRIzol reagent to extract RNA. RNA was reverse transcribed into complementary DNA with a reverse transcription kit according to the manufacturer’s instructions. q-PCR was performed using the following amplification conditions: denaturation at 94 °C for 30 s, 45 cycles of annealing at 60 °C for 15 s and extension at 72 °C for 10 s. The primers were synthesized by Shanghai Shenggong, and the Ct values of the target gene and the internal reference gene β-actin were determined. The relative expression level was calculated using the 2^−ΔΔCt^ method. The sequences of the primers are shown in Table 2.

**Table 2:** Primers used for q-PCR

Gene	Primer sequence (5’ to 3’)
*SDF-1****α (1)*** forward *SDF-1****α (1)*** reverse *SDF-1****α (2)*** forward *SDF-1****α (2)*** reverse *CD44* forward	TCTGAAAATCCTCAACACTCCA CAGGTACTCTTGGATCCACTTT CTCCTCTCGGATACCTCTTAGT CATGATCTCCACGATGTTCCTG CTCAAGTGCGAACCAGGACAGTG
*CD44* reverse	GTGCCAGGAGAGATGCCAAGATG
*CD90* forward	TGGTCAAGTGTGGCGGCATAAG
*CD90* reverse	GGAGGAGGGAGAGGGAAAGCAG
*Cyclin D1* forward *Cyclin D1* reverse	CGTATCTTACTTCAAGTGCGTG ATGGTCTCCTTCATCTTAGAGG
*RUNX2* forward	TTTAGGGCGCATTCCTCATC
*RUNX2* reverse	TGTCCTTGTGGATTAAAAGGACTTG
*OCN* forward	AGCAGGAGGGCAATAAGGTAGT
*OCN* reverse	ACCGTAGATGCGTTTGTAGGC
*β-Actin* forward	GGCTGTATTCCCCTCCATCG
*β-Actin* reverse	CCAGTTGGTAACAATGCCATGT

### Cell culture and grouping

OriCell™ C57BL/6 mouse BMSCs were purchased from Guangzhou Cyagen Company. These cells were inoculated in a culture flask at a density of 4.0 × 10^4^ cells/cm^2^ in complete medium (10% fetal bovine serum, glutamine, and 1% penicillin–streptomycin) at 37 °C with 5% CO_2_ in an incubator. Every 2–3 days, the medium was replaced with fresh complete medium. When the cells were 80–90% confluent, they were digested and passaged. All cells were used within 10 passages.

The cells were divided into the following 4 groups: the normal (N) group, SDF-1α overexpression (Lv-SDF-1α) group, empty vector (EV) group, and low SDF-1α expression (siRNA-SDF-1α) group. In this study, to avoid off-target effect and to make the conclusion more solid, two different RNA sequences were selected (SDF-1α (1) and SDF-1α (2)) to transfect BMSCs to make them highly expressed or low expressed. One of the sequences was selected for subsequent experiments.

### Cell transfection

Recombinant viral plasmids encoding lentiviral particles and their three auxiliary packaging original vector plasmids were prepared, subjected to high-purity endotoxin-free extraction, cotransfected into 293T cells with the transfection reagent RNAi-MATE (GenePharma, Shanghai, China), and replaced with complete culture media 6 h after transfection. After culture for 72 h, the cell supernatant enriched in lentiviral particles was collected and concentrated to obtain a high-titer lentiviral concentrate that was used to infect the target cells.

After three passages, the cells were collected, inoculated in a 24-well plate at a density of 1 × 10^4^ cells/mL, and incubated for 24 h at 37 °C with 5% CO_2_. Complete medium containing the lentiviral vector (GenePharma, Shanghai, China) was added to the 24-well plate at a multiplicity of infection (MOI) of 200, and the cells were incubated for 24 h. The next day, the medium was replaced with complete medium, and the cells were incubated for 4 days. Finally, the cells were observed under a microscope, and the data were recorded.

### Analysis of cell proliferation using a CCK-8 assay

Cells that were successfully transfected with the lentivirus and in good condition were collected to prepare a single cell suspension. Then, 100 µL of the cell suspension were added to a 96-well plate (6 replicate wells per group) and cultured for 5 days. Next, 10 µL of CCK-8 reagent (Dojindo Molecular Technologies, Inc.) were added to the 96-well plate, and the cells were incubated for 2 h. The OD was measured at 450 nm, and the results were recorded and analyzed.

### Cell cycle analysis

Cells in good condition after three passages were collected, and a 1 × 10^6^/mL single-cell suspension was prepared. One milliliter of the suspension was centrifuged at 1,500 rpm for 5 min, the supernatant was discarded, and the cells were fixed with cold ethanol and incubated for 12 h at 4 °C. The fixed cells were centrifuged at 1,500 rpm for 5 min and the supernatant was discarded. The cells were rinsed thoroughly with PBS, 0.1 mL of prepared staining solution was added, and the cells were incubated for 1 h in the dark. The number of cells in each phase of the cell cycle (G1, S, or G2/M phase) was determined.

### Osteogenic differentiation

Cells in good condition after three passages were collected to generate a single-cell suspension. The cells were inoculated in a 6-well plate coated with 0.1% gelatin, and 2 mL of complete medium were added. When the confluence reached 80–90%, the complete medium was discarded and replaced with osteogenic differentiation medium (containing 10% fetal bovine serum, 5 mM β-sodium glycerophosphate, 50 mg/mL ascorbic acid, and 10 nM dexamethasone; Cyagen, Guangzhou, China). Every 2–3 days, the medium was replaced with fresh osteogenic differentiation medium, and the shape and quantity of the cells were observed after three weeks.

### Alkaline phosphatase (ALP) staining

After 1 week of osteogenic differentiation, the medium was discarded, and the cells were rinsed thoroughly with PBS, fixed with 4% paraformaldehyde for 15 min, and rinsed again with PBS. The ALP incubation solution (Cyagen, Guangzhou, China) was added, and the cells were incubated for 20 min. Afterwards, cells were rinsed thoroughly, counterstained for 5 min, rinsed again, and observed.

### Alizarin Red S (ARS) staining

After 3 weeks of osteogenic differentiation, the culture medium was discarded, and the cells were washed with PBS and fixed with a 4% paraformaldehyde solution for 30 min. The fixative was discarded, the cells were washed with PBS, and ARS solution (Cyagen, Guangzhou, China) was added. The cells were incubated for 5 min and washed with PBS. The sections were observed under a microscope.

### Immunofluorescence staining of cells

Cells in good condition after three passages were collected and seeded on coverslips placed in a 24-well plate. The medium was replaced with fresh medium every 2 days. After 1 week, the medium was discarded and the cells were fixed with 4% paraformaldehyde for 20 min. The fixative was discarded, 0.5% Triton X-100 was added, and the cells were incubated at 37 °C for 15 min. Then, the cells were rinsed with PBS, and 50 µL of a goat serum working solution were added dropwise to the coverslips. The coverslips were incubated at 37 °C for 10 min and rinsed with PBS, and the primary antibody working solution was added dropwise. The coverslips were incubated at 4 °C for 12 h, rewarmed for 1 h on the next day, and incubated with the secondary antibody working solution at 37 °C for 2 h. Then, the cells were rinsed with PBS, DAPI was added dropwise, and the samples were observed under a microscope.

### Western blot analysis

Total protein was extracted from cells with RIPA buffer (Beyotime Institute of Biotechnology). The protein concentration was determined using the BCA method. Forty micrograms of protein were loaded in the wells of 10% sodium dodecyl sulfate polyacrylamide gels (SDS–PAGE), electrophoretically separated and transferred to a PVDF membrane. The PVDF membrane was blocked with 5% skim milk and incubated for two hours at room temperature. The blocked PVDF membrane was incubated with primary antibodies against SDF-1α (ab9797, 1:2000; Abcam), RUNX2 (ab23981, 1:1000; Abcam), OCN (DF12303, 1:500; Affinity), Cyclin D1 (ab134175, 1:10000, Abcam) and β-actin (bs-0061R, 1:5000; Bioss) at 4 °C overnight. The next day, a secondary antibody (1:5000) was added, and the membrane was incubated at room temperature for 2 h. Finally, the protein bands were visualized with ECL solution, and photos were captured.

### Statistical processing and analysis

In this study, GraphPad Prism 8 software was used to analyze the relevant experimental data, and the measurement data are presented as the means ± standard deviations. The difference in the means between groups was statistically analyzed using one-way ANOVA. P < 0.05 indicates a statistically significant difference.

## Results

### Increased expression of SDF-1α in OA

**Fig. 1:**
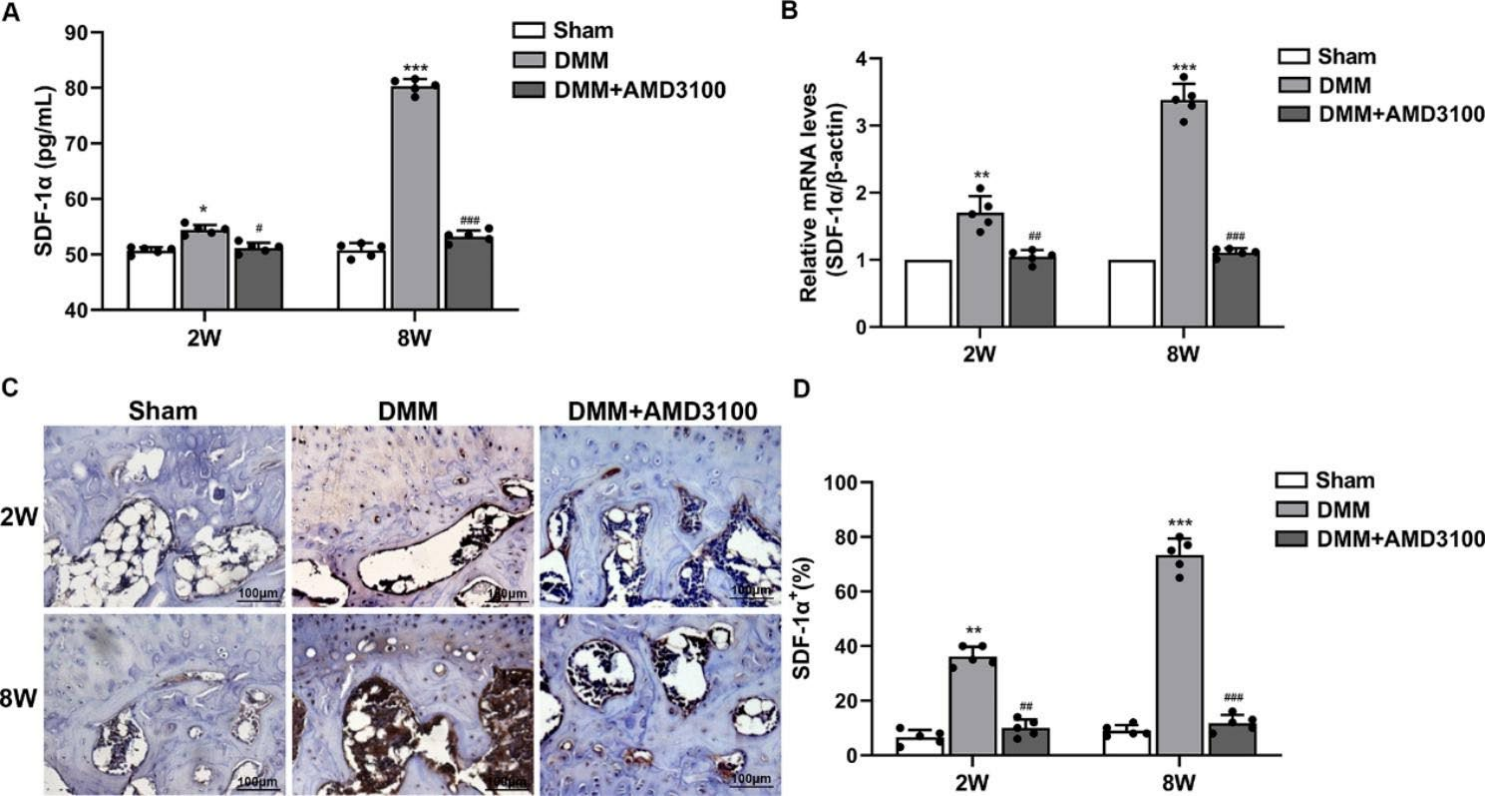
Expression of SDF-1α in a mouse model of OA. (**A**) Analysis of SDF-1α expression in peripheral blood using ELISA. (**B**) SDF-1α mRNA expression in subchondral bone at 2 and 8 weeks after the operation. (**C**) Immunohistochemical staining for SDF-1α in subchondral bone at 2 and 8 weeks after the operation. (**D**) Graph of SDF-1α expression in subchondral bone at 2 and 8 weeks after the operation. ^*^p < 0.05, ^**^p < 0.01, and ^***^p < 0.001 compared with the sham group

In this study, the peripheral blood and right knee joint specimens were collected from mice at 2 and 8 weeks to analyze changes in SDF-1α expression in the OA mouse model. The ELISA showed that compared with the sham group, SDF-1α expression in peripheral blood from the DMM group was slightly increased at 2 weeks and significantly increased at 8 weeks, while SDF-1α expression was significantly decreased in the DMM + AMD3100 group (Fig. 1A). The q-PCR results showed that compared with the sham group, SDF-1α mRNA expression was increased in subchondral bone from the DMM group to a certain extent at 2 weeks and significantly increased at 8 weeks, while in the DMM + AMD3100 group, SDF-1α expression was decreased (Fig. 1B). The immunohistochemical analysis revealed low SDF-1α expression in the sham-operated group but increases in SDF-1α intensity and the percentage of SDF-1α-positive cells beginning at 2 weeks and significant increases in these measures at 8 weeks in the DMM group. However, in the DMM + AMD3100 group, SDF-1α expression was decreased (Fig. 1C and D). Based on these results, SDF-1α expression increased with the aggravation of OA but decreased after an intraperitoneal injection of AMD3100.

### AMD3100 relieves the progression of OA

**Fig. 2:**
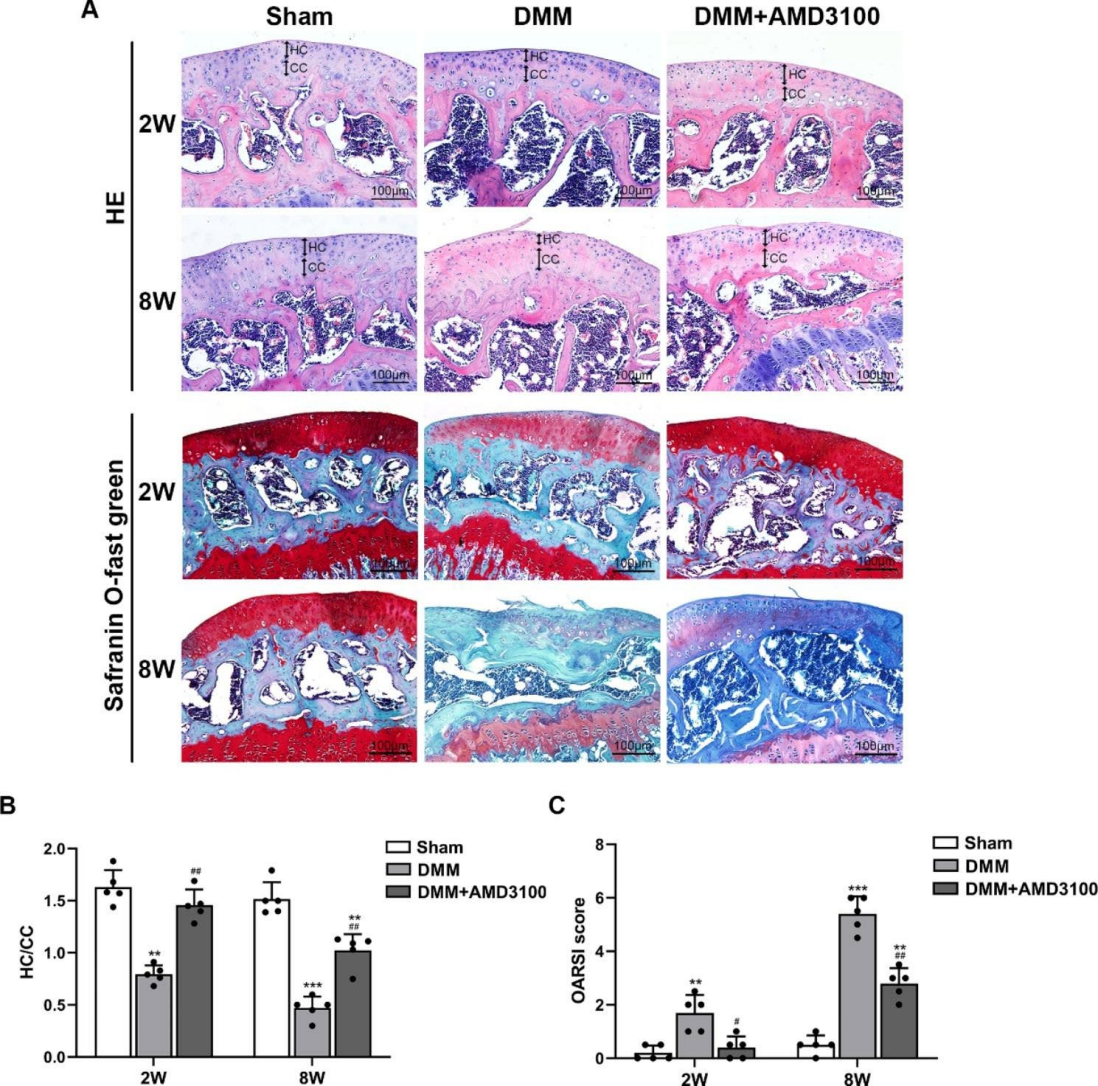
AMD3100 ameliorates OA progression. (**A**) HE staining and safranin-O fast green staining 2 and 8 weeks after surgery. (**B**) Quantitative analysis of the thicknesses of the HC and CC layers at 2 and 8 weeks after surgery. (**C**) OARSI scores were calculated to quantify the severity of articular cartilage destruction. ^**^p < 0.01 and ^***^p < 0.001 compared with the sham group; ^#^*p* < 0.05 and ^##^*p* < 0.01 compared with the DMM group

Mice were intraperitoneally injected with AMD3100 (an SDF-1α antagonist), and specimens were collected 2 and 8 weeks after surgery to verify the role of SDF-1α in the development of OA. The results of HE and safranin O-fast green staining showed that at 2 weeks, chondrocytes were hypertrophied, clustered, and vacuolated, proteoglycans were lost, safranin staining was lighter, the hyaline cartilage (HC) layer was thinner, and the calcified cartilage (CC) layer was thicker in the DMM group than in the sham group. At 8 weeks, the integrity of the surface cartilage was disrupted, the cartilage was severely degraded and worn, safranin staining was absent, the HC layer was thinner or even disappeared, the CC was thicker and hardened, and the subchondral bone was extensively exposed. In the DMM + AMD3100 group, joint degeneration caused by OA was significantly alleviated (Fig. 2A). The statistical analysis of HE staining revealed that the HC/CC layers in the DMM + AMD3100 group were thicker than those in the DMM group and thinner than those in the sham group (Fig. 2B). According to the OARSI scoring system, the severity of OA in the DMM + AMD3100 group was greater than that in the sham group and less than that in the DMM group (Fig. 2C). Based on this finding, SDF-1α may affect the occurrence and development of OA.

### AMD3100 relieves subchondral bone sclerosis in an OA model

**Fig. 3:**
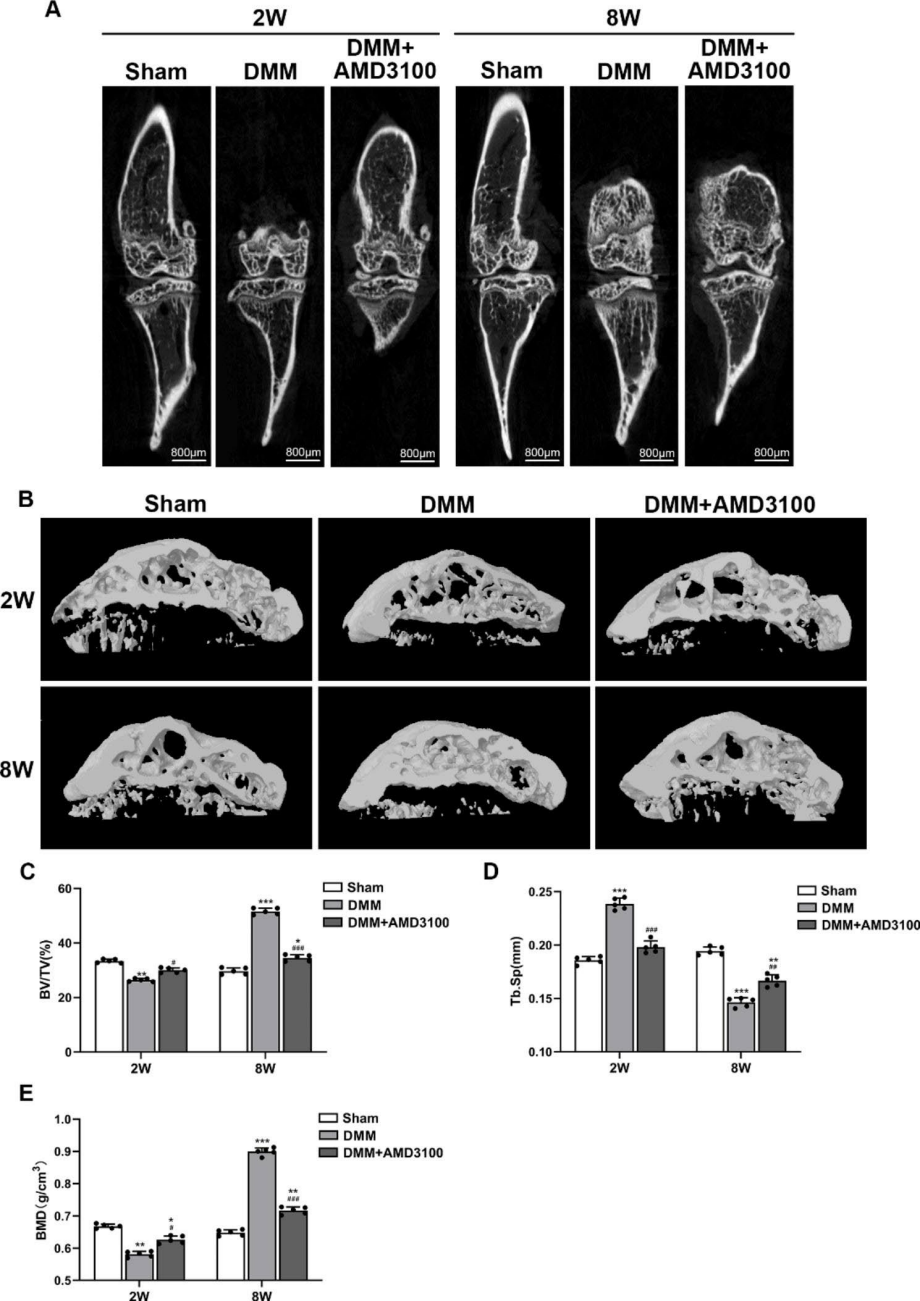
AMD3100 alleviates changes in subchondral bone in the OA model. (**A**) Changes in two-dimensional micro-CT images of the knee joint at 2 and 8 weeks after the operation. (**B**) Changes in 3-dimensional micro-CT images of the knee joint at 2 and 8 weeks after the operation. (**C**) Quantitative analysis of the BV/TV of subchondral bone. (**D**) Quantitative analysis of Tb. Sp of subchondral bone. (**E**) Quantitative analysis of subchondral bone BMD. ^*^p < 0.05, ^**^p < 0.01, and ^***^p < 0.001 compared with the sham group; ^#^*p* < 0.05, ^##^*p* < 0.01, and ^###^*p* < 0.001 compared with the DMM group

Micro-CT was performed to confirm the effect of AMD3100 (an SDF-1α antagonist) on subchondral bone in the OA model. At 2 weeks, a decrease in the mass of the subchondral bone was detected in the mouse tibia from the DMM group compared with that in the sham group. At 8 weeks, a significant increase in bone mass and bone sclerosis was observed (Fig. 3A and B). In the DMM + AMD3100 group, the expansion of the bone marrow cavity and bone mass were not observed at 2 weeks, and the increased bone mass and bone sclerosis were not observed at 8 weeks (Fig. 3A and B). The statistical results showed that BV/TV and BMD were decreased and Tb. Sp was increased in the DMM group compared with the sham group at 2 weeks, while the BV/TV and BMD were increased and Tb. Sp was decreased in the DMM group compared with the sham group at 8 weeks. In the DMM + AMD3100 group, these changes were alleviated (Fig. 3C, D and E). The experimental results described above show that antagonizing SDF-1α ameliorates the changes in subchondral bone in an OA model.

### AMD3100 reduces CD44 and CD90 expression in subchondral bone in the OA model

**Fig. 4:**
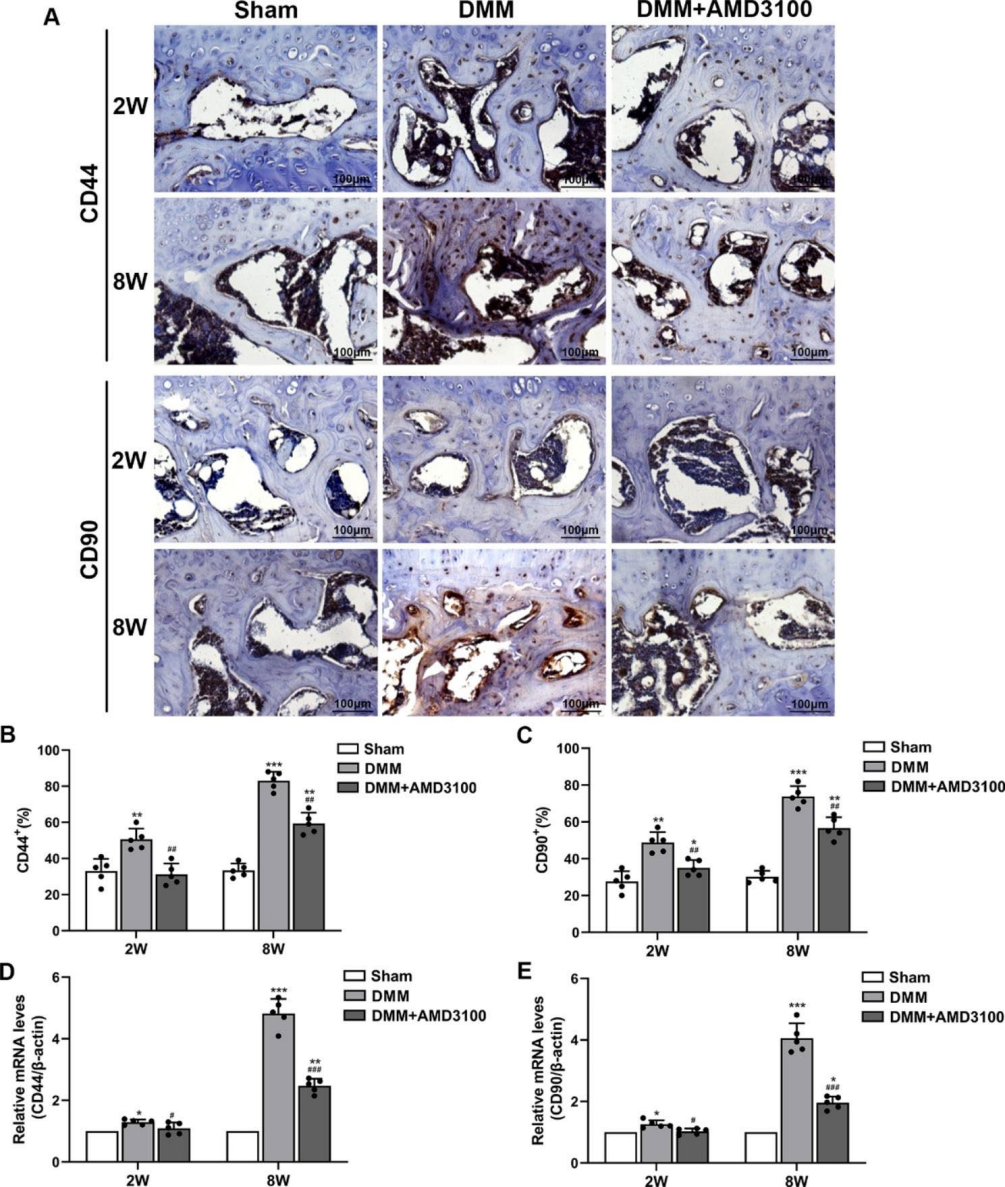
Expression of CD44 and CD90 in osteoarthritic subchondral bone. (**A**) Immunohistochemical staining for CD44 and CD90 in subchondral bone at 2 and 8 weeks after the operation. (**B**) Immunohistochemical analysis of CD44 expression in subchondral bone at 2 and 8 weeks after the operation. (**C**) Immunohistochemical analysis of CD90 expression in subchondral bone at 2 and 8 weeks after the operation. (**D**) CD44 mRNA expression in subchondral bone at 2 and 8 weeks after the operation. (**E**) CD90 mRNA expression in subchondral bone at 2 and 8 weeks after the operation. ^*^p < 0.05, ^**^p < 0.01, and ^***^p < 0.001 compared with the sham group; ^#^*p* < 0.05, ^##^*p* < 0.01, and ^###^*p* < 0.001 compared with the DMM group

CD44 and CD90 are specific markers expressed on the surface of BMSCs. In this study, immunohistochemistry and q-PCR were used to measure the expression of CD44 and CD90 in the subchondral bone of the mouse knee joint at 2 and 8 weeks as methods to evaluate the role of SDF-1α expressed on BMSCs in the progression of OA. Immunohistochemistry showed that compared with the sham group, the expression of CD44 and CD90 in subchondral bone in the DMM group began to increase at 2 weeks and was significantly increased at 8 weeks. In the DMM + AMD3100 group, these changes were alleviated (Fig. 4A, B and C). The q-PCR results showed increased levels of the CD44 and CD90 mRNAs in subchondral bone from the DMM group within 2 weeks that then continued to increase compared with the sham group. In the DMM + AMD3100 group, these changes were alleviated (Fig. 4D and E).

### AMD3100 reduces the expression of RUNX2 and OCN in subchondral bone in an OA model

**Fig. 5:**
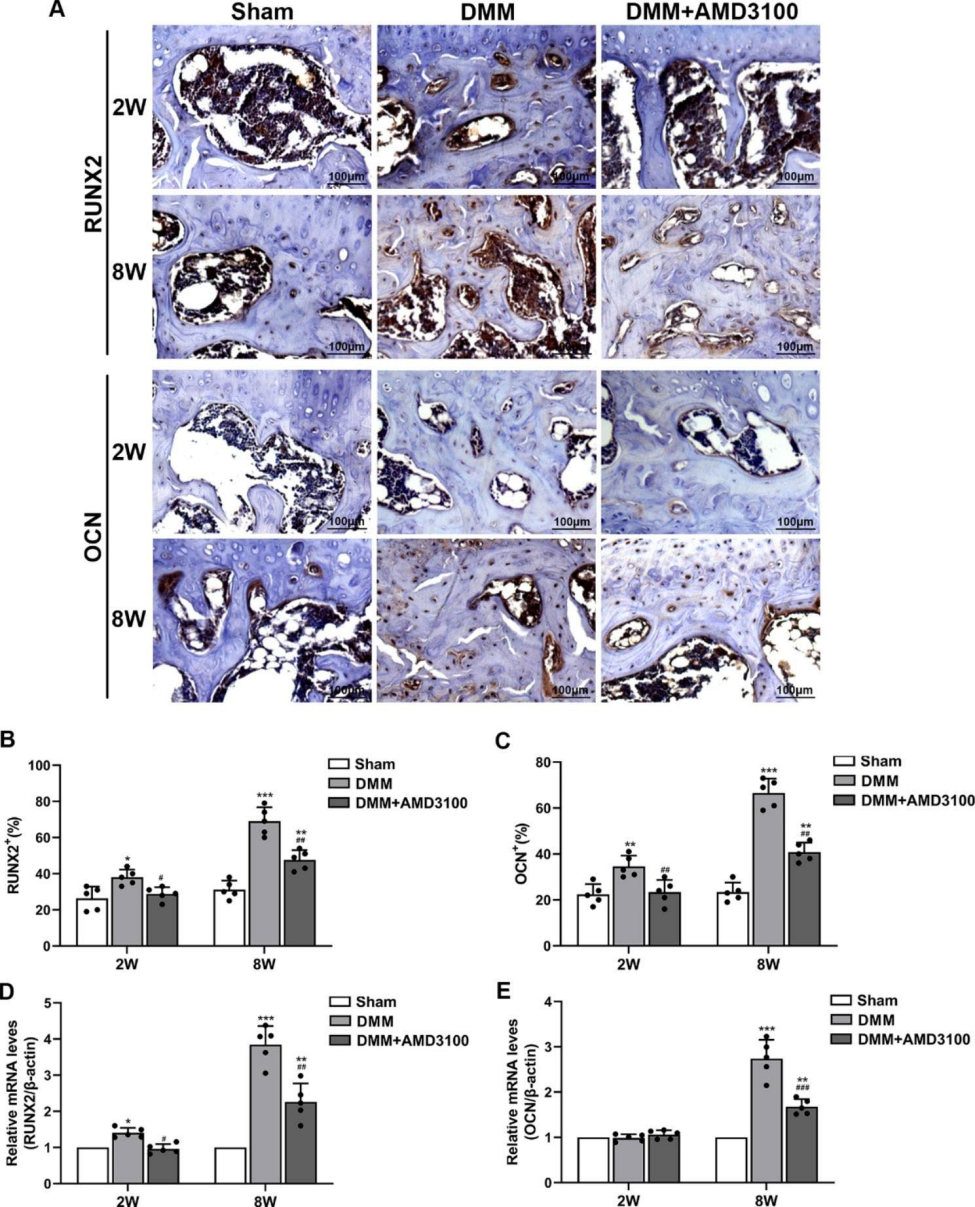
Expression of RUNX2 and OCN in osteoarthritic subchondral bone. (**A**) Immunohistochemical staining for RUNX2 and OCN in subchondral bone at 2 and 8 weeks after the operation. (**B**) Graph of the RUNX2 levels in subchondral bone at 2 and 8 weeks after the operation. (**C**) Graph of the OCN levels in subchondral bone at 2 and 8 weeks after the operation. (**D**) RUNX2 mRNA expression in subchondral bone at 2 and 8 weeks after the operation. (**E**) OCN mRNA expression in subchondral bone at 2 and 8 weeks after the operation. ^*^p < 0.05, ^**^p < 0.01, and ^***^p < 0.001 compared with the sham group; ^#^*p* < 0.05, ^##^*p* < 0.01, and ^###^*p* < 0.001 compared with the DMM group

RUNX2 and OCN are specific markers of osteogenic differentiation. In this study, immunohistochemistry and q-PCR were used to measure the expression of RUNX2 and OCN in the subchondral bone of the mouse knee joint at 2 and 8 weeks to assess the osteogenic ability of subchondral bone. Immunohistochemistry showed that compared with that in the sham group, RUNX2 expression in subchondral bone began to increase in the DMM group at 2 weeks and was significantly increased at 8 weeks. These changes were attenuated in the DMM + AMD3100 group (Fig. 5A and B). OCN expression was not significantly increased in subchondral bone from the DMM group at 2 weeks but was significantly increased at 8 weeks. The changes were attenuated in the DMM + AMD3100 group (Fig. 5A and C). The q-PCR results showed that the RUNX2 mRNA level was increased in the DMM group to a certain extent at 2 weeks and significantly increased at 8 weeks. In the DMM + AMD3100 group, these changes were reduced (Fig. 5D). OCN mRNA expression in the DMM group was not significantly altered at 2 weeks but was significantly increased at 8 weeks. These changes were attenuated in the DMM + AMD3100 group (Fig. 5E).

### BMSCs were successfully transfected with the lentivirus

**Fig. 6:**
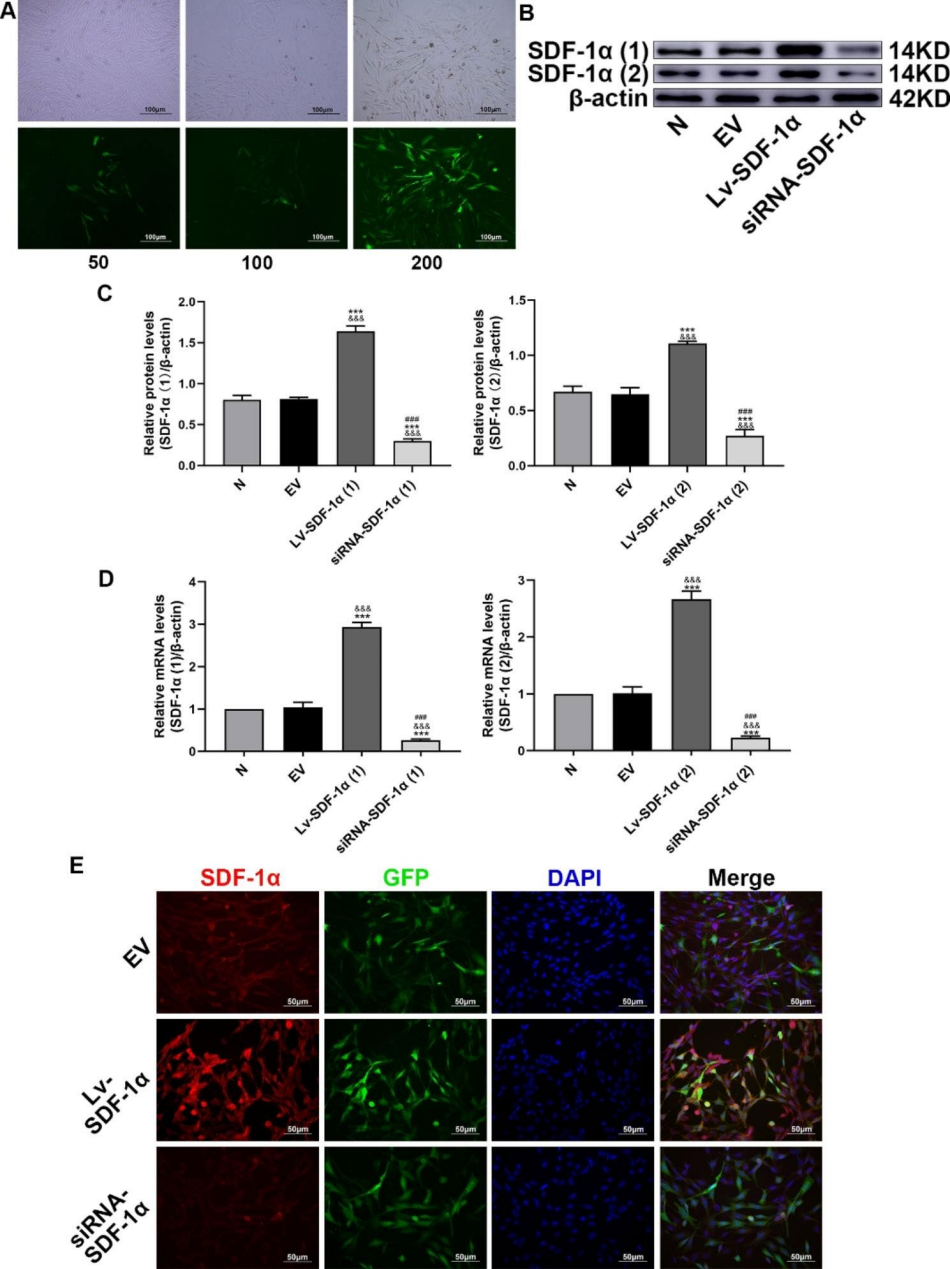
BMSCs were transfected with a lentivirus expressing the target gene. (**A**) Green fluorescence in BMSCs transfected with the lentivirus at different MOIs. (**B**) and (**C**) Western blot analysis of SDF-1α (1) and SDF-1α (2) protein expression and statistical analysis of the gray values; the group of blots was cropped from different parts of the same gel and the blots were cut prior to hybridisation with antibodies during blotting. (**D**) SDF-1α (1) and SDF-1α (2) mRNA expression. (**E**) SDF-1α expression was measured using immunofluorescence staining. ^&&&^*p* < 0.001 compared with the normal group, ^***^p < 0.001 compared with the EV group, and ^###^*p* < 0.001 compared with the Lv-SDF-1α group

In this study, BMSCs were transfected with the lentivirus at different MOIs. After four days of culture, green fluorescence was observed and analyzed, and the results were compared. At an MOI of 200, the green fluorescence intensity reached 80–90% (Fig. 6A). In subsequent experiments, BMSCs were transfected with lentivirus at an MOI of 200. BMSCs were transfected with the lentivirus expressing SDF-1α, EV, or siRNA-SDF-1α. Western blot analysis and q-PCR confirmed significantly higher SDF-1α expression in the Lv-SDF-1α group than that in the normal group. The expression level of SDF-1α in the siRNA-SDF-1α group was lower than that in the normal group and the EV group, but did not differ between the normal group and the EV group (Fig. 6B, C and D). In this study, the EV group was used as a negative control group. Immunofluorescence staining also confirmed that the fluorescence intensity of SDF-1α in the EV group was lower than that in the Lv-SDF-1α group but higher than that in the siRNA-SDF-1α group (Fig. 6E).

### SDF-1α promotes the proliferation of BMSCs

**Fig. 7:**
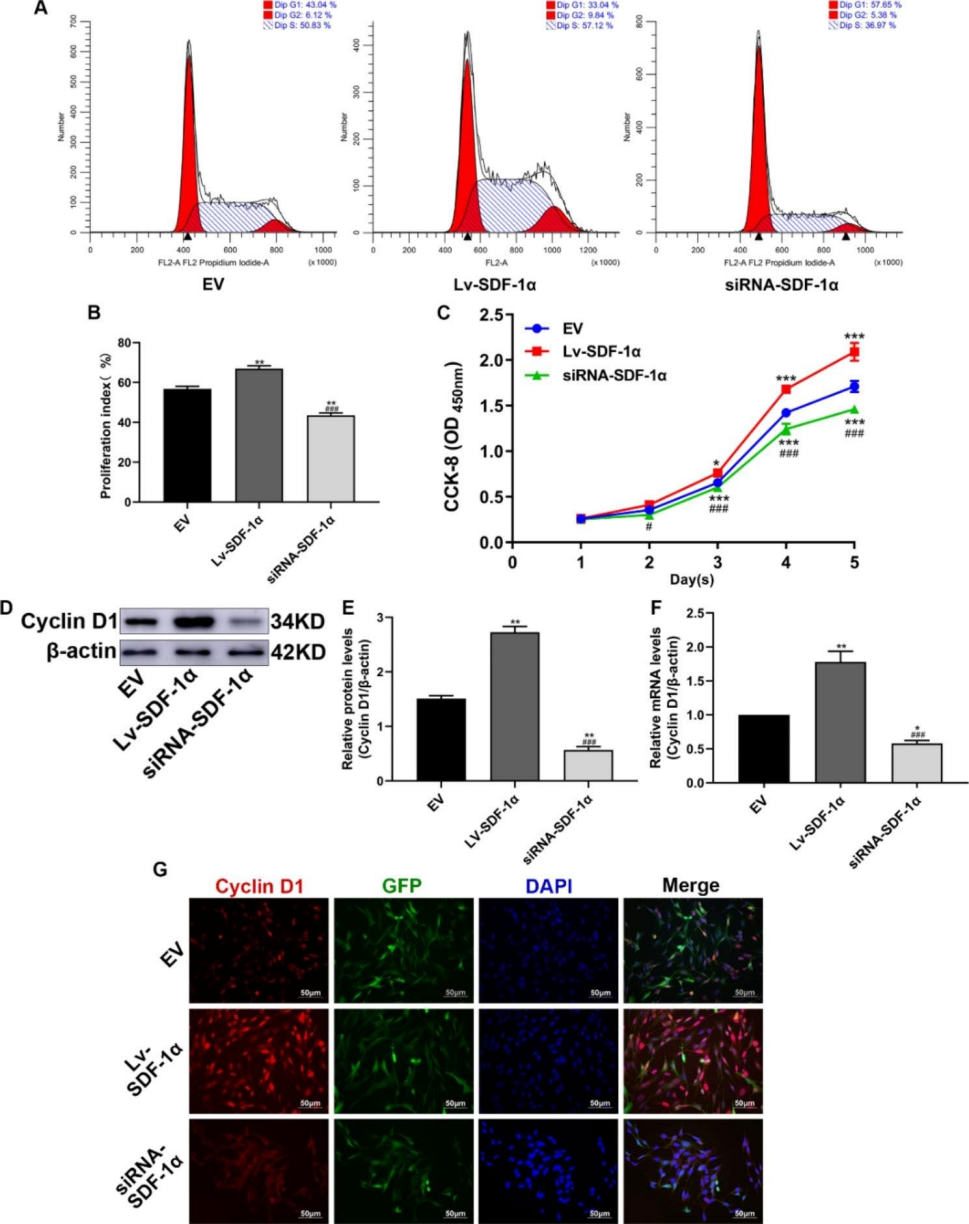
Effects of SDF-1α on BMSC proliferation. (**A**) Results of the cell cycle analysis. (**B**) Statistical analysis of the proliferation index determined from the cell cycle analysis. (**C**) Results of the CCK-8 assay. (**D**) and (**E**) Western blot analysis of cyclin D1 protein expression and statistical analysis of the gray values; the group of blots was cropped from different gels and the blots were cut prior to hybridisation with antibodies during blotting. (**F**) Cyclin D1 mRNA expression. (**G**) Cyclin D1 expression was measured using immunofluorescence staining. ^*^p < 0.05, ^**^p < 0.01, and ^***^p < 0.001 compared with the EV group; ^#^*p* < 0.05 and ^###^*p* < 0.001 compared with the Lv-SDF-1α group

In this study, a cell cycle analysis and a CCK-8 assay were used to evaluate the effect of SDF-1α on the proliferation of BMSCs. The results of the cell cycle analysis showed that G1 phase was longer in the EV group (48.62%) than that in the Lv-SDF-1α group (36.64%) and shorter than that in the siRNA-SDF-1α group (57.71%). G2 + S phase was shorter in the EV group (10.11 + 41.26%) than that in the Lv-SDF-1α group (11.03 + 52.33%) and longer than that in the siRNA-SDF-1α group (6.31 + 35.98%) (Fig. 7A). The cell proliferation index (G2 + S/G1 + G2 + S) was calculated to statistically analyze the results of the cell cycle analysis. BMSCs in the Lv-SDF-1α group proliferated faster than BMSCs in the EV group, while the proliferation of BMSCs in the siRNA-SDF-1α group was slower than that of BMSCs in the EV group (Fig. 7B). The results of the CCK-8 assay showed that the proliferation of BMSCs was significantly accelerated in the Lv-SDF-1α group but significantly delayed in the siRNA-SDF-1α group (Fig. 7C). Western blot analysis, q-PCR, and immunofluorescence staining showed that cyclin D1 was expressed at significantly higher levels in the Lv-SDF-1α group than in the EV group, and its expression in the siRNA-SDF-1α group was significantly lower than that in the EV group (Fig. 7D, E, F and G). Therefore, SDF-1α promotes the proliferation of BMSCs. When SDF-1α is expressed at low levels, the proliferation of BMSCs is delayed.

### SDF-1α promotes the osteogenic differentiation of BMSCs

**Fig. 8:**
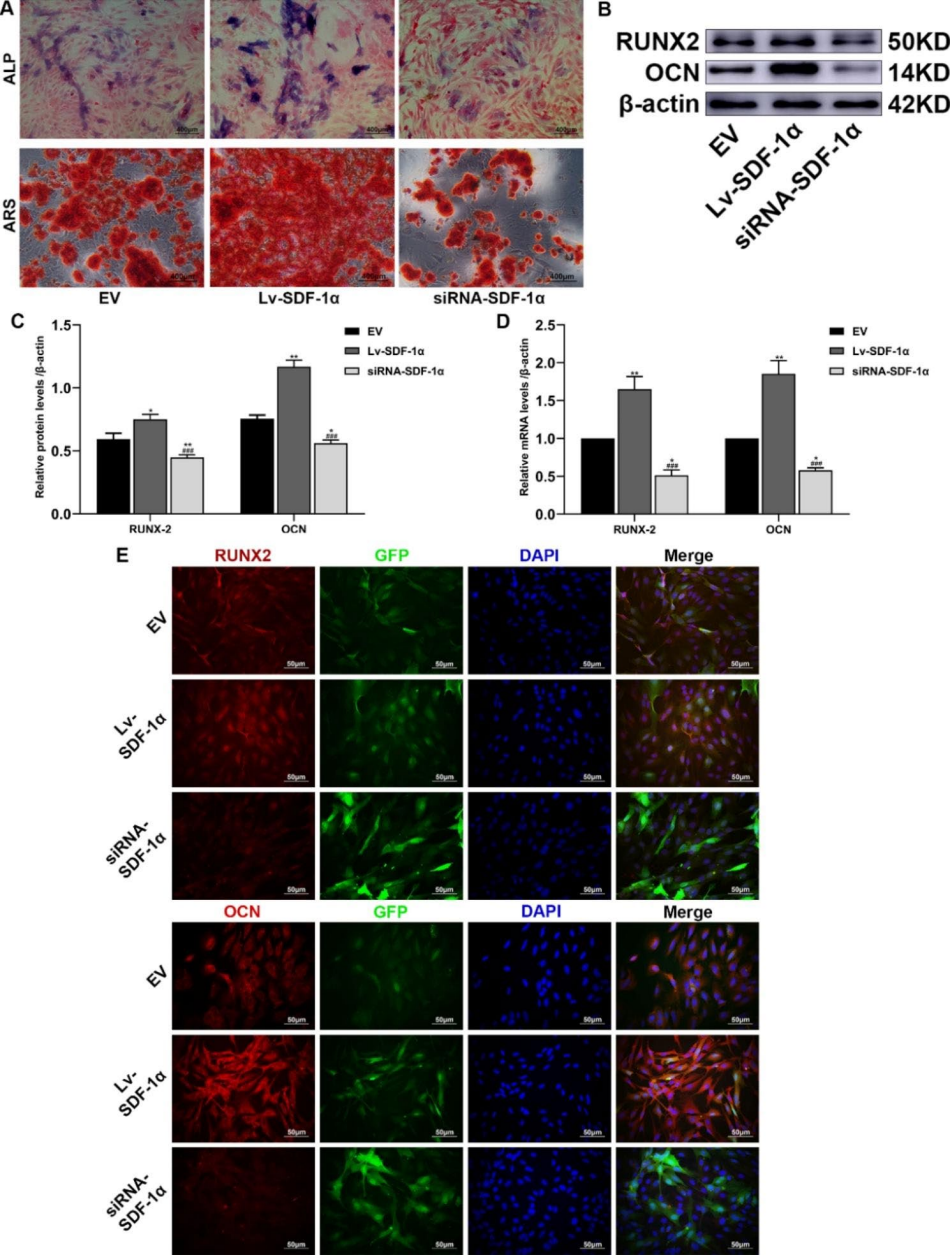
Effects of SDF-1α on the osteogenic differentiation of BMSCs. (**A**) ALP and ARS staining. (**B**) and (**C**) Western blot analyses of RUNX2 and OCN protein expression and statistical analysis of the gray values. The group of blots was cropped from different parts of the same gel and the blots were cut prior to hybridisation with antibodies during blotting. (**D**) RUNX2 and OCN mRNA expression. (**E**) The expression of RUNX2 and OCN was measured using immunofluorescence staining. ^*^p < 0.05 and ^**^p < 0.01 compared with the EV group; ^###^*p* < 0.001 compared with the Lv-SDF-1α group

In this study, ALP and ARS staining were performed to evaluate the effect of SDF-1α on the osteogenic differentiation of BMSCs. Stronger ALP staining was observed in the Lv-SDF-1α group than in the EV group and weaker staining was observed in the siRNA-SDF-1α group than in the EV group. The ARS staining results showed that the staining intensity of red mineralized nodules in the Lv-SDF-1α group was stronger than that in the EV group. Staining in the siRNA-SDF-1α group was weaker than that in the EV group (Fig. 8A). Western blot analysis, q-PCR, and immunofluorescence staining showed significantly higher RUNX2 and OCN expression levels in the Lv-SDF-1α group than those in the EV group, while the expression levels of these molecules in the siRNA-SDF-1α group were significantly lower than those in the EV group (Fig. 8B, C, D and E). Based on this result, high SDF-1α expression promotes the osteogenic differentiation of BMSCs, while low SDF-1α expression inhibits the osteogenic differentiation of BMSCs.

## Discussion

OA, a common chronic degenerative joint disease that often leads to chronic pain, functional impairment and disability in middle-aged and elderly people, imposes a very severe social burden and has become a major public health problem [14]. However, the pathology of OA is very complex and has not been completely elucidated. In recent years, an increasing number of clinical studies have shown that SDF-1α may play a certain role in the progression of OA [15], but its specific mechanism has not been conclusively determined.

Because the tissue damage caused by DMM is insignificant, the disease progresses slowly; mild changes were observed 2 weeks after surgery, and marked changes were observed at 8 weeks, mainly in the tibial plateau [16]. These changes are similar to the pathological changes observed in humans; thus, this animal model of the disease has been used to study its pathology [17]. Therefore, in this study, an OA model was constructed by DMM. In this study, the SDF-1α levels in the peripheral blood and subchondral bone of the knee joints of mice subjected to DMM started to increase at 2 weeks according to ELISA, immunohistochemistry and q-PCR and were significantly increased by 8 weeks. In addition, in this study, AMD3100 was intraperitoneally injected into mice to antagonize SDF-1α, and the severity of DMM-induced OA was alleviated, according to HE staining and safranin O-fast green staining. These results suggest that SDF-1α plays an important role in the progression of OA and that SDF-1α expression continues to increase during the progression of the disease, consistent with previous reports [18]. SDF-1α is expressed in joint synovium and cartilage [19]. In the present study, SDF-1α was continuously expressed and played a role in subchondral bone.

Relevant studies have found that subchondral bone abnormalities are closely related to OA [20]. Under stress conditions, abnormal bone remodeling occurs first in the subchondral bone, resulting in increased subchondral bone formation and bone sclerosis [21]. When stress occurs again, the subchondral bone is unable to absorb the pressure and buffer the shock, resulting in an increase in the pressure load on the cartilage and cartilage damage [22]. Micro-CT showed that in the DMM group, the BV/TV and BMD of subchondral bone were decreased, and Tb. Sp was increased at 2 weeks, while the BV/TV and BMD were increased and Tb. Sp was decreased at 8 weeks. These results indicate that abnormal bone remodeling occurs in the subchondral bone of the OA model, with bone resorption occurring in the early stage and bone formation occurring in the late stage. Furthermore, SDF-1α was antagonized with AMD3100, the microstructure of the subchondral bone was improved, the abnormal remodeling of subchondral bone was alleviated, bone resorption was inhibited in the early stage, and bone formation was inhibited in the late stage. Relevant studies have shown that RUNX2 plays an important role in the formation and reconstruction of bone tissue and exerts a certain regulatory effect on transcription [23]. OCN is a specific marker of advanced osteogenic differentiation and bone mineralization [24]. In the present study, immunohistochemistry and q-PCR showed that RUNX2 expression in subchondral bone in the DMM group was increased at 2 weeks and significantly increased at 8 weeks, while OCN expression was not significantly increased at 2 weeks but was significantly increased at 8 weeks. After SDF-1α antagonism, the expression of RUNX2 and OCN in subchondral bone was decreased. Based on these results, SDF-1α may affect the progression of OA by regulating abnormal remodeling of subchondral bone. The in vitro experiments showed that SDF-1α promotes osteoclast differentiation, which may be the mechanism by which bone resorption is inhibited after the early antagonism of SDF-1α. However, few studies have investigated the mechanism by which late subchondral bone formation is inhibited after SDF-1α antagonism.

Studies have shown that CD44 and CD90 are specific markers expressed on the surface of BMSCs [25]. In this study, immunohistochemistry and q-PCR showed that CD44 and CD90 expression in subchondral bone in the DMM group started to increase at 2 weeks and were significantly increased at 8 weeks. After SDF-1α antagonism, CD44 and CD90 expression decreased. The number of BMSCs in subchondral bone was increased in the OA model but was decreased after SDF-1α antagonism. Some in vivo and in vitro experiments have confirmed that the SDF-1α receptor CXCR4 is expressed on the surface of BMSCs. The expression of SDF-1α is upregulated in injured liver, heart, skin and other tissues, leading to the recruitment and migration of CXCR4-expressing BMSCs from peripheral blood to the injury site [26]. The results of this study indicate that in the OA model, the expression of SDF-1α is increased, which in turn promotes an increase in the number of BMSCs in subchondral bone.

BMSCs constitute a class of stem cells that exhibit multidirectional differentiation potential, and most BMSCs are distributed in the bone marrow [27]. In response to certain stimuli, BMSCs differentiate into osteoblasts, thereby maintaining the balance of bone metabolism [28]. SDF-1α is derived from a class of small-molecule proteins secreted by immune and nonimmune cells that plays key roles in autoimmunity, allergy, inflammation, and cell proliferation and differentiation [29]. Accumulating evidence suggests that SDF-1α in BMSCs plays an important role in the treatment of bone defects, bone repair, and OA [30]. However, the underlying molecular mechanism remains unclear. As shown in the present study, SDF-1α accelerate the proliferation and osteogenic differentiation of BMSCs.

Lentiviral vectors are vectors derived from an immunodeficient virus that infect cells [31]. Upon foreign gene invasion, lentiviruses are reverse transcribed into the genome to achieve durable and stable expression, and lentiviral vectors do not generate an effective cellular immune response [32]. In this study, BMSCs were transfected with lentivirus expressing SDF-1α, EV, or SDF-1α-targeted siRNA. Western blot analysis, q-PCR and immunofluorescence staining confirmed that the cells were successfully transfected with and stably and continuously expressed the lentivirus.

In this study, a CCK-8 assay and cell cycle analysis were performed to evaluate BMSC proliferation. The CCK-8 assay is mainly used to test cell proliferation and is highly sensitive [33]. A cell cycle analysis is commonly used to evaluate adherent or suspended cells, and the numbers of cells in G1, S, and G2 phases are measured using flow cytometry. The proliferation index (G2 + S/G1 + G2 + S%) was calculated to assess the cell proliferation rate [34]. Cyclin D1 is a cyclin that allows cells to rapidly progress through the cell cycle, thereby accelerating cell proliferation [35]. The absorbance in the CCK-8 assay, the proliferation index and cyclin D1 expression were significantly increased in the Lv-SDF-1a group, indicating that SDF-1α overexpression promotes the proliferation of BMSCs. Studies have shown that endothelial cells self-proliferate through autocrine SDF-1α signaling and recruit mesenchymal stem cells to promote bone formation, consistent with the results of the present study [36].

ALP staining and ARS staining are mainly used to analyze the osteogenic differentiation of BMSCs. ALP is a phosphomonoester hydrolase, most of which is produced by osteocytes, and it is often used as an indicator of early osteogenic differentiation [37]. ARS reacts with calcium salts deposited on the cell surface to produce dark red compounds that are often used as markers of advanced osteogenic differentiation [38]. RUNX2 plays an important role in osteoblast differentiation, is closely related to bone formation and is an important transcription factor [39]. OCN is a vitamin K-dependent calcium-binding protein that plays an important role in regulating bone calcium metabolism and is a biochemical marker of advanced bone formation according to the staining results described in this study [40]. Compared to the control group, ALP staining and ARS staining were stronger and RUNX2 and OCN expression were higher in the Lv-SDF-1α group, while ALP staining and ARS staining were weaker and RUNX2 and OCN expression were lower in the siRNA-SDF-1α group. Based on these results, SDF-1α overexpression promotes the osteogenic differentiation of BMSCs.

## Conclusions

The in vivo and in vitro experiments performed in this study revealed that SDF-1α was expressed at high levels in subchondral bone in an OA model and that by promoting the proliferation and osteogenic differentiation of BMSCs, SDF-1 hardened subchondral bone and aggravated the progression of OA, providing clinically significant evidence for the pathogenesis of osteoarthritis.

## Electronic supplementary material

Below is the link to the electronic supplementary material.

Supplementary Material 1

## Data Availability

All data generated or analyzed during this study are included in this published article.

## Abbreviations

OA: Osteoarthritis
BMSCs: Bone marrow mesenchymal stem cells
SDF-1α: Stromal cell-derived factor-1α
DMM: Destabilization of the medial meniscus
HE: Hematoxylin and eosin
ELISA: Enzyme-linked immunosorbent assay
q-PCR: Quantitative real-time polymerase chain reaction
EV: Empty vector
ALP: Alkaline phosphatase
ARS: Alizarin red S
RUNX2: Runt-related transcription factor 2
OCN: Osteocalcin
