# Supplementary material for: SDF-1α promotes subchondral bone sclerosis and aggravates osteoarthritis by regulating the proliferation and osteogenic differentiation of bone marrow mesenchymal stem cells
Source: BMC Musculoskelet Disord. 2023 Apr 10;24:275. doi: 10.1186/s12891-023-06366-1 (PMC10088262; doi:10.1186/s12891-023-06366-1)
Supplement: Supplementary file 1 — Supplementary Material 1 [file 12891_2023_6366_MOESM1_ESM.pdf]

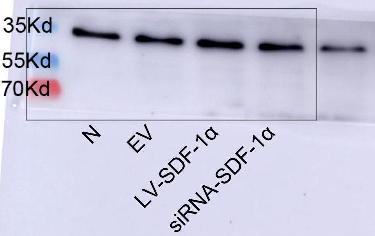

Figure 6 β-actin 42Kd

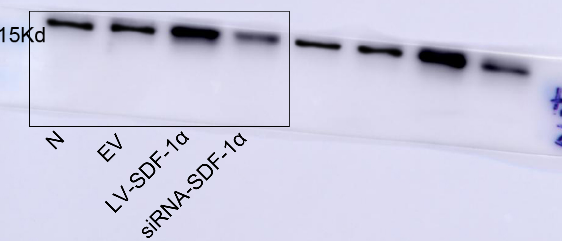

Figure 6 SDF-1α (1) 14Kd

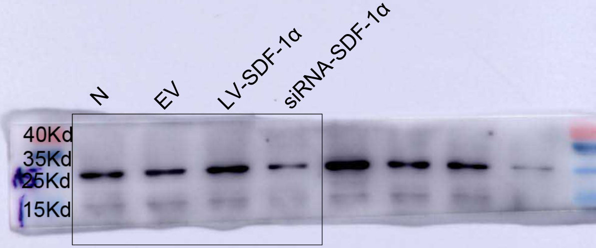

Figure 6 SDF-1α (2) 14 Kd

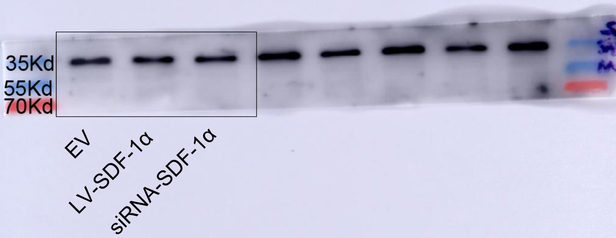

Figure 7 β-actin 42Kd

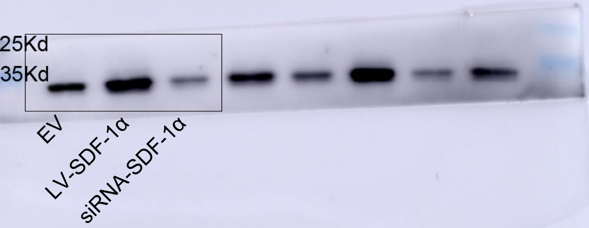

Figure 7 Cyclin D1 34Kd

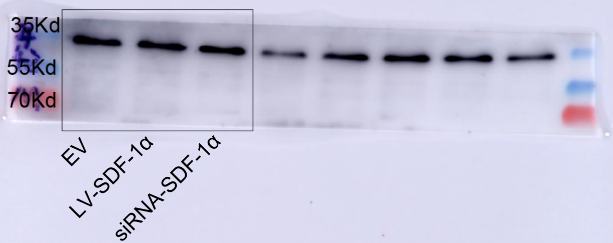

Figure 8 β-actin 42Kd

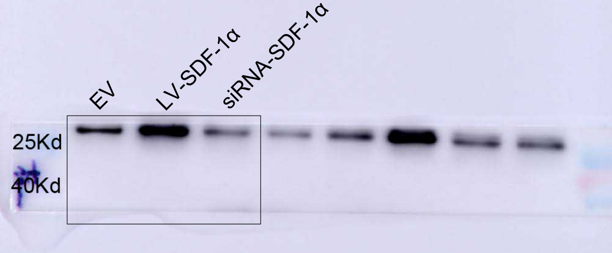

Figure 8 OCN 14Kd

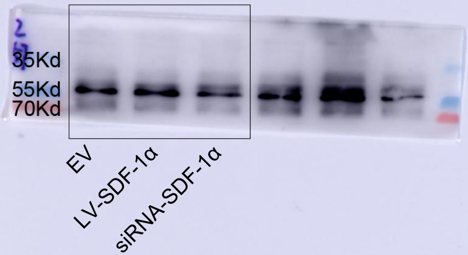

Figure 8 RUNX2 57Kd
